# Comparison of Early Clinical and Long-Term Oncological Outcomes of Laparoscopic Versus Converted Rectal Cancer Resection: A Retrospective Cohort Study

**DOI:** 10.7759/cureus.65086

**Published:** 2024-07-22

**Authors:** Ulas Aday, Abdulkadir Akbaş, Ferdi Bayrak, Zehra Şekho, Azat Közgün, Murat Sevmis, Abdullah Oğuz

**Affiliations:** 1 Gastrointestinal Surgery, Dicle University, Diyarbakir, TUR; 2 General Surgery, Dicle University School of Medicine, Diyarbakır, TUR

**Keywords:** minimal access surgery, oncological outcomes, conversion, laparoscopy, rectal cancer

## Abstract

Aim

The effects of conversion to open surgery during laparoscopic resection in rectal cancer on perioperative clinical and long-term oncological outcomes are still controversial. This study aimed to evaluate and compare the impact of conversion to laparoscopic resection for rectal cancer on perioperative and long-term oncological outcomes.

Material and methods

Between January 2019 and December 2023, 84 consecutive patients who underwent curative surgery for rectal cancer at a single academic center were evaluated retrospectively. Patients were classified and compared as the laparoscopic (LAP-G) and converted (CONV-G) groups. Perioperative, pathological, and long-term oncological outcomes were compared.

Results

Of the 84 consecutive patients included, 18 were converted to open surgery, leading to a 21.4% conversion rate. Intraoperative blood loss was higher in CONV-G (180 ml vs. 80 ml, p<0.001), but early clinical outcomes were similar in both groups. The median follow-up period was 23.5 (range 3-65) and 30.5 (range 6-61) months in the LAP-G and CONV-G, respectively, and recurrence occurred in 11 (16.7%) and 3 (16.6%) patients, respectively. Three-year overall survival was 96.9% and 89.4% (p=0.609) and 3-year disease-free survival was 92.4% and 83.3% (p=0.881) in LAP-G and CONV-G, respectively, and the results were similar.

Conclusion

Conversion from laparoscopic rectal resection to open surgery does not have a significant negative impact on morbidity and long-term oncological outcomes.

## Introduction

The standardization of total mesorectal excision (TME) and neoadjuvant chemoradiotherapy significantly improved oncological outcomes in rectal cancer surgery [1]. Colorectal cancer surgery centers are increasingly using laparoscopic surgery due to its early clinical advantages. Although there may be concerns about oncological outcomes in the early periods, with well-standardized surgery, long-term oncological outcomes similar to the open procedure can be achieved [2,3]. Conversion rates for laparoscopic rectal cancer surgery have been reported between 17% and 29% [4,5]. The reasons for conversion from laparoscopy to open are usually multifactorial and may include patient, surgeon, or tumor-related factors. Obesity, technical skill level, distal location, narrow pelvis, combined resections, and locally advanced or bulky tumors are known risk factors for conversion to open surgery [6-8].

The perioperative and oncological outcomes of laparoscopic versus converted surgery are not homogeneous in the literature. Although some studies have reported that the duration of surgery, blood loss, postoperative complications, and hospital stay increase and have a negative effect on long-term oncological outcomes [6,8-12], other studies have reported that clinical and oncological outcomes are similar [13-18]. Prospective randomized clinical trials are difficult because conversion cannot be predicted, so the issue will continue to be controversial. In this retrospective cohort study, we aimed to compare not only the early perioperative clinical outcomes but also the overall survival (OS) and disease-free survival (DFS) outcomes of laparoscopic completed and converted rectal cancer resection.

## Materials and methods

Study design and patients

Retrospective analyses of patients who underwent curative resection for rectal cancer between January 2019 and December 2023 were obtained from a prospectively maintained clinical database. The study was completed at the Department of General Surgery, School of Medicine, Dicle University (Diyarbakır, Turkey). The laparoscopic surgery program for colorectal cancer cases has been initiated in our department since 2010 and currently, 80-100 procedures are performed annually. Laboratory parameters, thoracic-abdominopelvic computed tomography, and pelvic magnetic resonance imaging were performed in all patients whose diagnosis of rectal adenocarcinoma was confirmed by endoscopic evaluation, and a treatment plan was created by a multidisciplinary tumor board.

Ethical considerations

After institutional review board approval, all patients who underwent curative resection for rectal cancer between January 2019 and December 2023 were reviewed (approval number: 2024-186). Written informed consent was obtained from all patients included in the study and the standards of the Declaration of Helsinki (1964) were followed.

Study criteria

**Figure 1:**
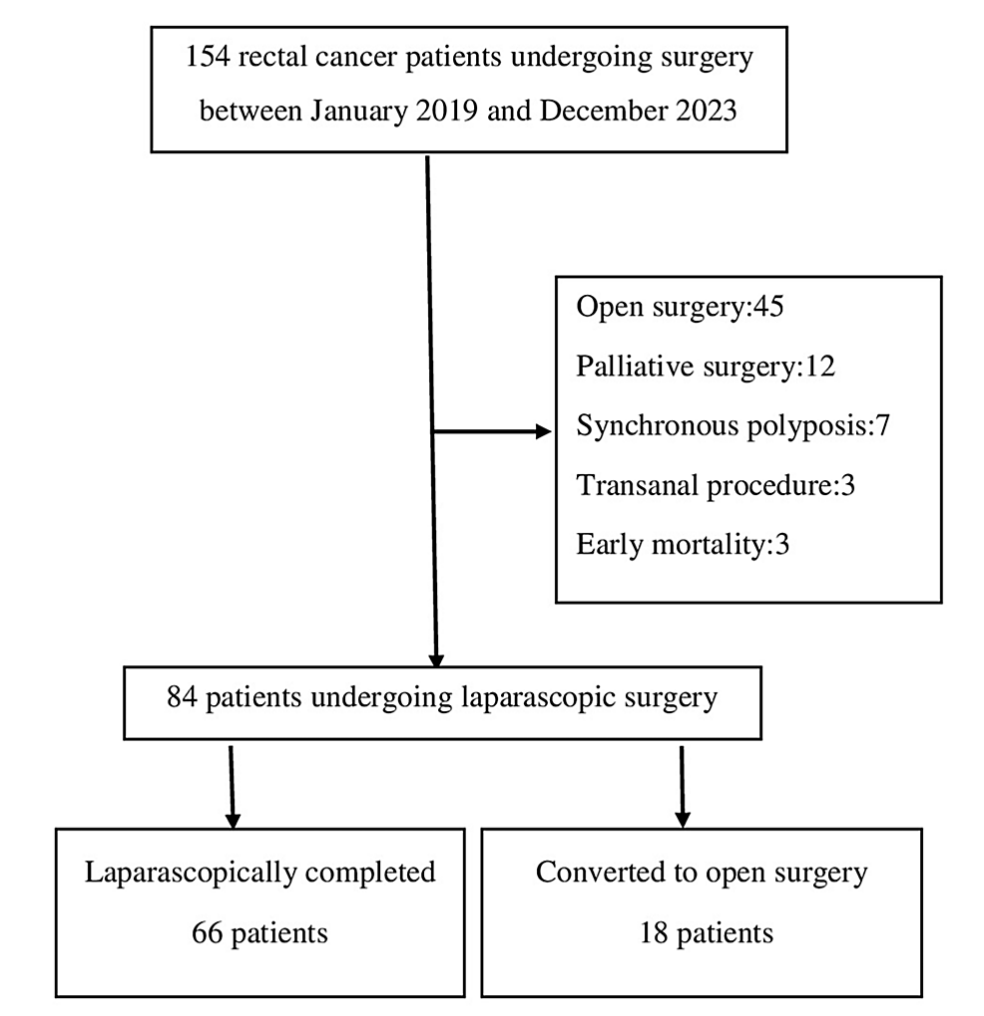
Flow chart of the study

Patients younger than 18 years of age, missing medical records, with open-onset surgery, proctocolectomy for concomitant polyposis, simultaneous multiple organ resection, palliative surgical procedure, early mortality (first 90 days), and endoscopic or transanal organ-sparing approaches were excluded from the analysis (Figure 1). The decision to convert to laparotomy was at the discretion of the surgeon and was defined as an additional incision that was not planned for any reason. Patients were classified and compared as laparoscopic (LAP-G) and converted (CONV-G) groups.

Procedure

Curative surgery was performed 8-12 weeks after the completion of radiotherapy. Sphincter‑preserving surgery was performed with complete splenic flexure and left‑colon mobilization in a medial‑to‑lateral and superior‑to‑inferior fashion. After high ligation of the inferior mesenteric vessels, following the avascular plan of the mesorectal plane, standard TME was performed by descending to the level of the levator muscle with sharp dissection [19,20]. In metastatic cases, R0 was achieved by synchronous or staged resection in accordance with the recommendation of the multidisciplinary tumor board.

Assessments

Demographic data (age, gender, comorbid conditions, body mass index (BMI)), Eastern Cooperative Oncology Group Performance Status (ECOG PS), pre-treatment carcino embryogenic antigen (CEA) level, length of hospital stay, operative data (surgical procedure, operation time, blood loss, blood transfusion, stoma status), and pathological data were investigated. Tumor location was defined as the lower rectum (distal tumor margin less than 5 cm from the anal verge), middle rectum (5-10 cm from the anal verge), and upper rectum (10-15 cm from the anal verge) according to the anal verge distance. Complications that developed in the first postoperative 90 days were categorized according to the Clavien-Dindo classification and grade ≥3 was considered major morbidity [21]. All patients were followed up every three months in the first year, every six months in the next 2 years, and then annually thereafter. Recurrence was defined according to radiological or biopsy results (when necessary) or surgical exploration. DFS was defined as the time between the date of surgery and the time when recurrence was first detected. OS was defined as the time from the date of diagnosis to the date of death. For patients with no evidence of any event, the last follow-up data constituted the terminal record.

Statistical analysis

Data were analyzed using IBM SPSS Statistics for Windows ver. 21.0 (IBM Corp., Armonk, NY, USA). Continuous variables were expressed as mean ± standard deviations or median with ranges. Categorical variables were expressed as frequencies (number) and percentages (%). Continuous variables were compared using the independent samples t-test and categorical variables were compared using the chi-square test or Fisher's exact test. The OS was estimated using the Kaplan-Meier method and the outcomes were compared using the log-rank test. A P value of less than 0.05 was considered significant.

## Results

One hundred and fifty-four consecutive patients underwent surgery for rectal cancer. Sixty-seven patients who did not meet the study protocol criteria (45 open-onset surgery, 12 palliative procedures, 7 synchronous polyposis, 3 trans-anal organ-sparing surgery, and 3 early mortality) were excluded from the study, and 84 patients were included in the final analysis (Figure 1). Of the 84 patients included in the study, 18 were converted to open surgery (CONV-G), leading to a conversion rate of 21.4%. Sixty-six patients completed laparoscopic surgery (LAP-G). T4 or bulky tumors (11; 61.1%), radiotherapy-related dense adhesion (4; 22.2%), tumor perforation (1), postoperative adhesion (1), and cardiac arrhythmia (1) were the reasons for conversion surgery.

The demographic, operative, and clinical characteristics of the groups are summarized in Table 1. The remaining characteristics were similar except that the CEA level was above normal (p=0.008) and intraoperative blood loss was higher in CONV-G (p <0.001). Neoadjuvant treatment was administered to 52 (78.8%) and 11 (61.1%) in LAP-G and CONV-G, respectively. Sphincter-sparing surgery was performed in 59 (89.4%) patients in LAP-G and 16 (88.9%) in CON-G. Synchronous resectable metastases were present in three patients (2 liver, 1 lung) in LAP-G and one patient (liver) in CONV-G. R0 was achieved in three patients with liver metastases, one synchronously and two patients with staged surgery. One patient with oligometastasis in the lung was treated with staged surgery. Anastomotic leakage was seen in 6 (16.7%) patients in CONV-G and 3 (9.1%) patients in LAP-G. Although it was more common in CONV-G, the difference was not statistically significant (p=0.395). The length of hospital stay was 8 days (range 5-27) in LAP-G and 11.5 days (range 5-27) in CONV-G, and although it was higher in CONV-G, it was not statistically significant (p=0.067).

**Table 1:** Demographic, clinical, and perioperative characteristics BMI, body mass index; CEA, carcinoembryonic antigen; ECOG PS, Cooperative Oncology Group Performance Status; SD, standard deviation

Characteristics	LAP-G	CONV-G	P value
	(n = 66); n (%)	(n = 18); n (%)	
Median age (years, range)	57 (23-84)	54.5 (32-80)	0.957
Gender			0.76
Males	38 (57.6%)	9 (50.0%)	
Females	28 (42.4%)	9 (50.0%)	
BMI kg/m^2^ (mean ± SD)	25.7±3.60	24.6±3.10	0.214
ECOG PS			0.284
0	23 (34.8%)	7 (38.9%)	
1	39 (59.1%)	8 (44.4%)	
2	4 (6.1%)	3 (16.7%)	
Tumor location			0.155
Upper rectum	20 (30.3%)	7 (38.9%)	
Mid rectum	27 (40.9%)	3 (16.7%)	
Lower rectum	19 (28.8%)	8 (44.4%)	
CEA level (ng/L)			0.008
<5	57 (86.4%)	10 (55.6%)	
≥5	9 (13.6%)	8 (44.4%)	
Neoadjuvant therapy	52 (78.8%)	11 (61.1%)	0.137
Surgical procedure			1
Sphincter-sparing	59 (89.4%)	16 (88.9%)	
Abdominoperineal resection	7 (10.6%)	2 (11.1%)	
Stoma formation	59 (89.4%)	17 (94.5%)	0.665
Operative time (min; mean (SD))	251.4±67.4	296.1±98.4	0.084
Blood loss (ml; median (range))	80 (25-320)	180 (80-290)	<0.001
Intraoperative transfusion required	3 (4.5%)	0 (0.0%)	
Clavien-Dindo			0.052
<3	60 (90.9%)	13 (72.2%)	
≥3	6 (9.1%)	5 (27.8%)	
Anastomotic leak	6 (9.1%)	3 (16.7%)	0.395
Length of hospital stay (days)	8 (5-27)	11.5 (5-27)	0.067

In the pathological evaluation of the resection specimens, there was no difference between the number of resected lymph nodes, positive margin rates, and ypTNM stage distributions (Table 2).

**Table 2:** Pathologic and tumor-related variables

Characteristics	LAP-G	CONV-G	P value
	(n = 66) n (%)	(n = 18) n (%)	
T category			0.333
0-1-2	29 (43.9%)	5 (27.8%)	
3-4	37 (56.1%)	13 (72.2%)	
Lymph node status			0.447
pN0	55 (83.3%)	17 (94.4%)	
pN+	11 (16.7%)	1 (5.6%)	
Positive resection margins	1 (1.5%)	0 (0.0%)	1
Lymph nodes (median (range))	12.5 (3-50)	14 (2-51)	0.913
Degree of differentiation			0.147
Well	36 (64.3%)	14 (82.4%)	
Moderately	9 (16.1%)	3 (17.6%)	
Poorly	11 (19.6%)	0 (0.0%)	
Unknown	10 (15.2%)	1 (5.6%)	
Lymphovascular invasion	2 (3.0%)	0 (0.0%)	1
Perineural invasion	3 (4.5%)	1 (5.6%)	1
ypTNM stage			0.363
0 (pCR)	9 (13.6%)	1 (5.6%)	
I	17 (25.8%)	2 (11.1%)	
II	27 (40.9%)	12 (66.7%)	
III	10 (15.2%)	2 (11.1%)	
IV	3 (4.5%)	1 (5.6%)	

**Figure 2:**
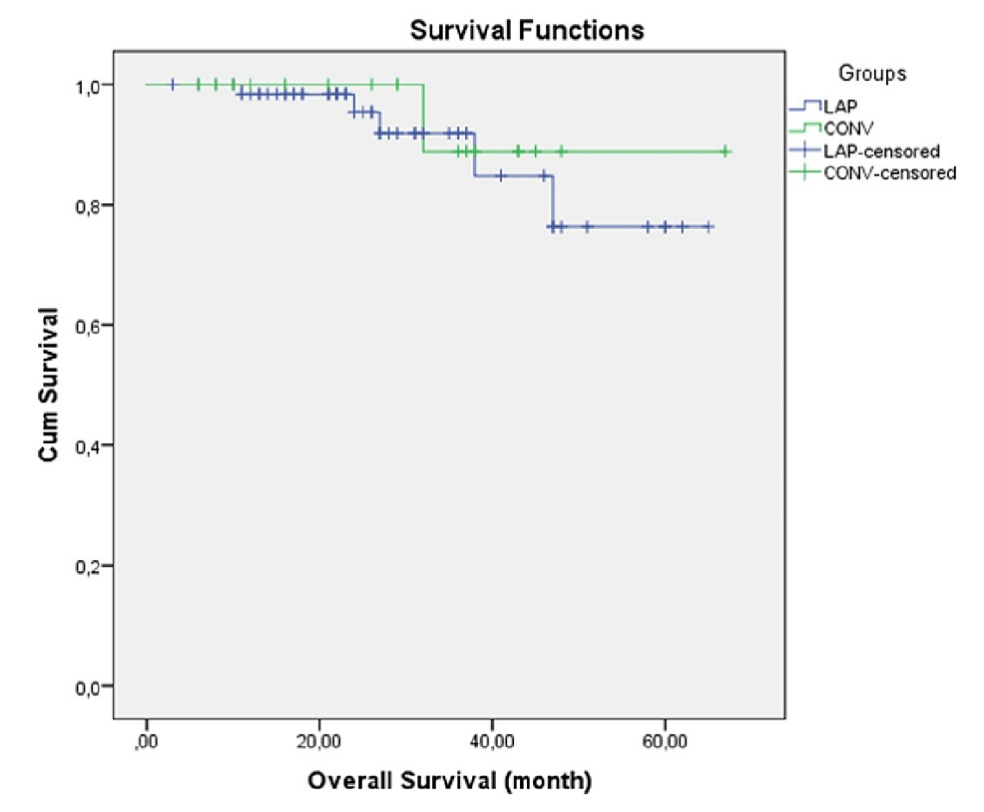
Kaplan-Meier overall survival curve in the laparoscopic and conversion groups (p = 0.609; log-rank test)

**Figure 3:**
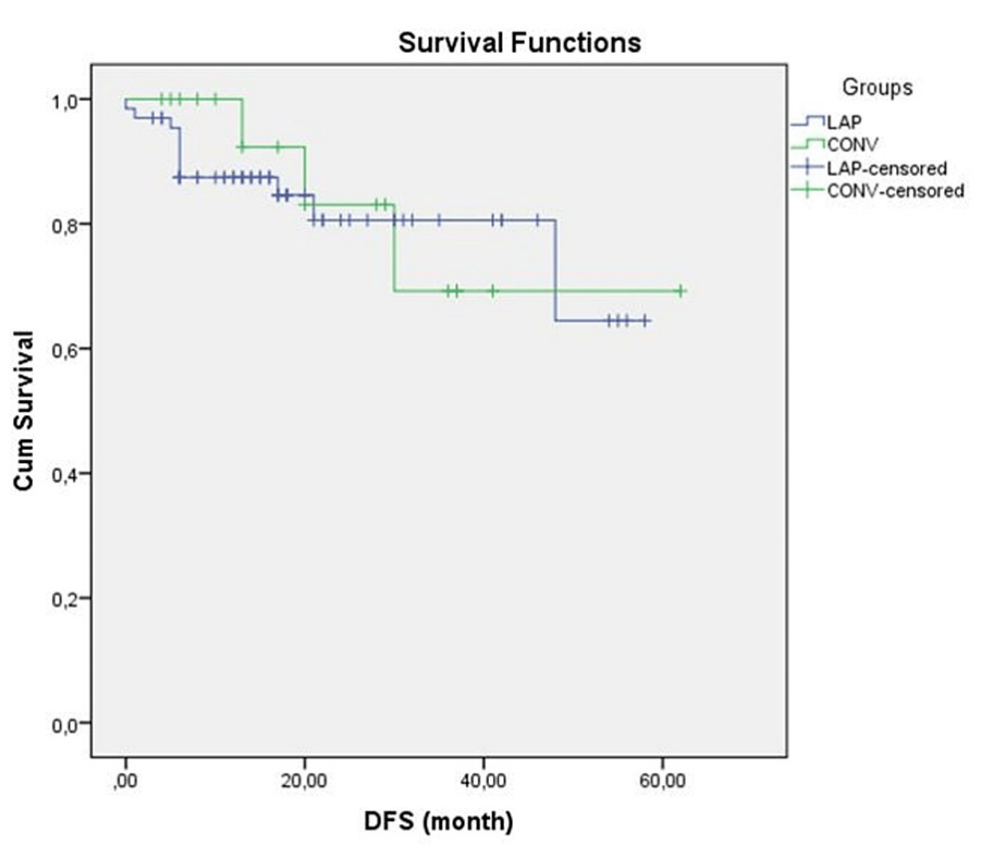
Kaplan-Meier disease-free survival curve in the laparoscopic and conversion groups (p=0.881; log-rank test)

Long-term oncologic outcomes are shown in Table 3. The median follow-up period was 23.5 (range 3-65) and 30.5 (range 6-61) months in the laparoscopic and conversion groups, respectively, and recurrence occurred in 11 (16.7%) and 3 (16.6%) patients, respectively. Three-year overall survival was 64 (96.9%) and 17 (89.4%) (p=0.609), and 3-year disease-free survival was 92.4% and 83.3% (p=0.881) in LAP-G and CONV-G, respectively, and the results were similar (Figures 2-3).

**Table 3:** Long-term oncological outcomes

Variable	LAP-G	CONV-G	P value
	(n = 66) n (%)	(n = 18) n (%)	
Median follow-up: years (range)	23.5 (3-65)	30.5 (6-61)	0.392
Overall recurrence	11 (16.7%)	3 (16.7%)	1
Disease-related mortality	5 (7.6%)	1 (5.6%)	1
3-year overall survival	64 (96.9%)	17 (89.4%)	0.609
3-year disease-free survival	61 (92.4%)	15 (83.3%)	0.881

## Discussion

Large-scale randomized controlled trials in the last decade have shown that laparoscopic surgery gives better short-term results than laparotomy for rectal cancer, does not increase perioperative complication rates, and pathological outcomes are similar [22-24]. In addition, a similar long-term prognosis between laparoscopic surgery and laparotomy in terms of recurrence-free survival has been reported and the oncological safety of both techniques has been demonstrated [2,25,26]. Laparoscopic rectal cancer surgery is more difficult than for colon cancer and conversion to laparotomy rates of 16% in the well-designed randomized controlled COLOR II trial [27], 9% in the ALaCaRT trial [24], and 2% in the recent EnSSURE trial [8] were reported. Conversion rates are expected to be lower in specialized centers with certified surgical teams. The reasons for conversion from laparoscopy to open are multifactorial. Conversion to laparotomy is not itself a complication; the clinician's first priority is patient safety and completion of the surgical procedure in accordance with oncological principles [8,11,16,17]. A locally advanced tumor is an important reason for conversion from laparoscopy to open. Timing of conversion is important in these cases. Laparoscopic long manipulation may cause perforation, bleeding, and tumor cell seeding. Therefore, if the conversion decision is made early, it may prevent these undesirable outcomes and reduce morbidity [15,28]. In this study, the conversion rate from laparoscopy was 18 (21.4%), which is high and consistent with the literature. T4 or bulky tumors and dense adhesion associated with radiotherapy are the two most important reasons. R0 resection was achieved in all patients who were converted to laparotomy, which is very important in terms of achieving the targeted oncological results.

It is emphasized in the literature that conversion in rectal cancer surgery has a negative effect on early clinical results. A recent meta-analysis published by Finochi et al. [6] reported that anastomotic leakage, wound infection, and morbidity increased in conversion surgery, but mortality outcomes were similar. Similarly, a review of 10,845 patients by Gouvas and colleagues [12] found that the duration of operation, the length of hospital stay, and other complications were higher in the conversion group than in the laparoscopic group. However, in retrospective studies including large series of patients, they found that completion of the surgical procedure with conversion did not increase early mortality and morbidity [4,15,29]. When the perioperative results of our study were evaluated, it was observed that the blood loss measured in CONV-G was higher (180 ml versus 80 ml, p<0.001). Operative time (296 vs. 251 minutes), major complication rate 5 (27.8%) in CONV-G vs. 6 (9.12%) in LAP-G, anastomotic leakage rate 3 (16.7%) vs. 6 (9.1%), and length of hospital stay (11.5 vs. 8 days) were higher in the CONV-G group but not statistically significant. The limited number of patients may have contributed to the lack of a significant difference in the analyses.

In this study, although the follow-up period was shorter, we found that the oncological results were similar. Three-year DFS and OS were 92.2% vs 83.3% and 96.9% vs 89.4% in LAP-G and CONV-G, respectively, and the difference was not significant. Although five-year OS and DFS were slightly better in LAP-G (92.4% vs. 89.4% and 90.4% vs. 83.3%, respectively), the difference was not significant in the analysis (p=0.609, p=0.881). Perioperative undesirable conditions result in a greater systemic inflammatory response in conversion surgery, which is thought to negatively affect long-term oncological outcomes [30]. In a study examining 57 cases undergoing conversion, distal tumor location and conversion were identified as factors that negatively affected survival [16]. Although the study results regarding oncological outcomes are heterogeneous, they tend to be worse [11]. Conversion to open surgery is usually not the surgeon's preference. Intraoperative complications (e.g., bleeding, perforation), large tumor size, distal location, obesity, narrow pelvis, and locally advanced tumor are the predominant factors. All these factors negatively affect oncological outcomes and survival, therefore conversion to laparotomy alone may not be an indicator of poor prognosis. Patient safety and completion of optimal oncological surgery are the priority and early conversion decisions will positively affect perioperative and long-term outcomes [8,11,12,15].

There are some limitations in our study. Although the data were collected prospectively, they are retrospective and include results from a single academic center. Second, the number of cases is small and the follow-up period is not sufficient for long-term oncological outcomes. Third, those who underwent open surgery were not included in the analysis. However, we think that the results given in two homogeneous groups with complete data are important.

## Conclusions

In conclusion, this study showed an increase in blood loss when converting from minimally invasive to open surgery, but no significant difference was observed, although other major complications, such as anastomotic leakage and length of hospital stay, were higher. Although long-term oncologic outcomes were slightly worse in the conversion group, the results were similar.
